# How to Buy Textiles Cheaply

**Published:** 1916-02-12

**Authors:** W. Wall

**Affiliations:** St. John Ambulance Brigade. Walcot, Wembley


### THE EDITOR'S LETTER-BOX.

### HOSPITAL EQUIPMENT IN WAR-TIME.

### How to buy Textiles Cheaply.

Sir,—Being interested in hospital work, your correspondent's letter is somewhat disquieting. But, although raw materials have very much advanced in price, the manufacture of the same into articles of use for hospitals, etc., has not yet reached 100 per cent. over pre-war times. For instance, I find on inquiry that the business houses who hold large stocks, such as Maple's, are still prepared to sell sheets at 7s. 11d. per pair that before the war were 6s. 11d., and many other articles in like proportion. Yet I think the warning of "J. A. H." is timely, and should be noted by all concerned in either renewing stocks or fitting up new hospitals, as the tendency in prices is decidedly to increase rather than diminish. Apologising for taking up your valuable space, I beg to remain, yours faithfully, W. Wall,

St. John Ambulance Brigade.

Walcot, Wembley, February 7, 1916.

